# Association of Inflammatory and Oxidative Status Markers with Metabolic Syndrome and Its Components in 40-to-45-Year-Old Females: A Cross-Sectional Study

**DOI:** 10.3390/antiox12061221

**Published:** 2023-06-05

**Authors:** Katarína Šebeková, Marta Staruchová, Csilla Mišľanová, Aurélia Líšková, Mira Horváthová, Jana Tulinská, Miroslava Lehotská Mikušová, Michaela Szabová, Radana Gurecká, Ivana Koborová, Melinda Csongová, Tamás Tábi, Éva Szökö, Katarína Volkovová

**Affiliations:** 1 Institute of Molecular Biomedicine, Medical Faculty, Comenius University in Bratislava, 83303 Bratislava, Slovakia; radana.kollarova@gmail.com (R.G.); koborova@gmail.com (I.K.); melinda.csongova@gmail.com (M.C.); 2 Institute of Biology, Medical Faculty, Slovak Medical University in Bratislava, 83303 Bratislava, Slovakia; marta.staruchova@szu.sk (M.S.); katarina.volkovova@szu.sk (K.V.); 3 Institute of Nutrition, Faculty of Nursing and Medical Professional Studies, Slovak Medical University in Bratislava, 83303 Bratislava, Slovakia; csilla.mislanova@szu.sk; 4 Department of Immunology and Immunotoxicology, Slovak Medical University in Bratislava, 83303 Bratislava, Slovakia; aurelia.liskova@szu.sk (A.L.); mira.horvathova@szu.sk (M.H.); jana.tulinska@szu.sk (J.T.); miroslava.mikusova@szu.sk (M.L.M.); michaela.szabova@szu.sk (M.S.); 5 Institute of Medical Physics, Biophysics, Informatics and Telemedicine, Faculty of Medicine, Comenius University in Bratislava, 83303 Bratislava, Slovakia; 6 Department of Pharmacodynamics, Faculty of Pharmacy, Semmelweis University, 1085 Budapest, Hungary; tabi.tamas@pharma.semmelweis-uni.hu (T.T.); eva.szoko@pharma.semmelweis-uni.hu (É.S.)

**Keywords:** cardiometabolic risk markers, uric acid, bilirubin, antioxidative enzymes, sRAGE, cellular adhesion molecules

## Abstract

Oxidative stress and sterile inflammation play roles in the induction and maintenance of metabolic syndrome (MetS). This study cohort included 170 females aged 40 to 45 years who were categorized according to the presentation of MetS components (e.g., central obesity, insulin resistance, atherogenic dyslipidemia, and elevated systolic blood pressure) as controls not presenting a single component (n = 43), those with pre-MetS displaying one to two components (n = 70), and females manifesting MetS, e.g., ≥3 components (n = 53). We analyzed the trends of seventeen oxidative and nine inflammatory status markers across three clinical categories. A multivariate regression of selected oxidative status and inflammatory markers on the components of MetS was performed. Markers of oxidative damage (malondialdehyde and advanced-glycation-end-products-associated fluorescence of plasma) were similar across the groups. Healthy controls displayed lower uricemia and higher bilirubinemia than females with MetS; and lower leukocyte counts, concentrations of C-reactive protein, interleukine-6, and higher levels of carotenoids/lipids and soluble receptors for advanced glycation end-products than those with pre-MetS and MetS. In multivariate regression models, levels of C-reactive protein, uric acid, and interleukine-6 were consistently associated with MetS components, although the impacts of single markers differed. Our data suggest that a proinflammatory imbalance precedes the manifestation of MetS, while an imbalance of oxidative status accompanies overt MetS. Further studies are needed to elucidate whether determining markers beyond traditional ones could help improve the prognosis of subjects at an early stage of MetS.

## 1. Introduction

Central obesity, insulin resistance, atherogenic dyslipidemia, and elevated blood pressure (BP) increase the risk of the manifestation of chronic conditions. Their clustering confers a higher risk when compared with the sum of the risks posed by individual components. Thus, the concurrent presentation of at least three out of the five causally interconnected cardiometabolic risk factors (e.g., central obesity, elevated BP, fasting plasma glucose (FPG), triacylglycerols (TAG), and low levels of high-density lipoprotein cholesterol (HDL-C)) has been termed metabolic syndrome (MetS) [1,2,3,4,5]. MetS confers about a 5-fold increase in the risk of type 2 diabetes mellitus, a 2-fold increase in the risk for cardiovascular morbidity and mortality, and a 1.5-fold increase in the risk for all-cause mortality [6,7].

Although markers of inflammation and oxidative stress are not part of any definition of MetS, the coexistence of a persistent link and a tight interrelationship between low-grade inflammation [8,9,10,11] and an imbalance in oxidative status [12,13] with MetS is generally accepted. In individuals with MetS, sterile inflammation and oxidative stress are important factors increasing the risk of manifesting cardiovascular diseases [9,14]. 

Adipose tissue, particularly visceral adipose tissue, is a potent endocrine organ that produces adipokines, cytokines, and other regulatory molecules [14]. Bioactive molecules released from enlarged visceral adipocytes into systemic circulation promote sterile inflammation, insulin resistance, endothelial cell dysfunction, hypertension, and atherosclerosis [15,16,17,18]. Moreover, they induce the local recruitment of innate and adaptive immune cells expressing an inflammatory phenotype and secreting proinflammatory cytokines [14,19,20,21]. However, sterile inflammation characterizes hypertension, insulin resistance, and atherogenic dyslipidemia, even in the absence of obesity [18,22,23].

Oxidative stress reflects an increased load of pro-oxidants resulting from their overproduction, ineffective detoxification, and/or a reduction in savaging antioxidants, eventually leading to irreversible damage to proteins, lipids, and nucleic acids [24,25]. 

The mitochondrial electron transport system, oxidases, oxygenases, and uncoupled nitric oxide synthase represent physiologically relevant sources of reactive oxygen species (ROS) [26]. Under homeostasis, ROS function as essential signaling molecules. In MetS, increased levels of free fatty acids, insufficient HDL antioxidant capacity, inflammatory cytokines, adipokines, angiotensin II, mitochondrial dysfunction, endoplasmatic reticulum stress, and low antioxidant levels contribute to elevated oxidative stress and are simultaneously associated with a chronic inflammatory state [8]. To combat oxidative damage, organisms developed endogenous enzymatic and non-enzymatic antioxidants which work together with exogenous (dietary) antioxidants. 

Several clinical studies focused on the association of MetS or a number of its components with inflammatory markers (e.g., leukocyte counts, C-reactive protein (CRP), interleukine-6 (IL-6), IL-10, tumor necrosis factor-α (TNF-α), etc.) [10,27,28,29,30,31,32,33,34,35,36,37] or soluble adhesion molecules (e.g., intercellular adhesion molecule-1 (sICAM-1), sE-selectin, vascular adhesion protein-1 (sVAP-1), etc.) [37,38,39,40,41,42].

Plasma levels of oxidative defense markers—albumin, bilirubin, and uric acid [27,28,29,43,44,45,46,47]—and the activities of antioxidative enzymes, e.g., superoxide dismutase (SOD), glutathione peroxidase (GPx), and catalase (CAT) [10,28,32,46,48,49,50,51,52], have been compared between MetS-free subjects and those presenting with MetS. Similarly, differences in the concentrations of oxidatively modified substrates, which are used in clinical practice as proxy measures of oxidative stress, such as malondialdehyde (MDA) [10,27,32,43,44,48,49], advanced glycation end products (AGEs), and their soluble receptor (sRAGE) [53,54,55,56], have been investigated.

Generally, the previously mentioned studies monitored only a few indicators at a time, and their results are often contradictory. Discrepancies might reflect methodology, e.g., the cohorts differ by age, ethnicity, and lifestyle factors, sex is not always taken as a biological variable, or the classification of MetS and the prevalence and severity of its components differ.

In this analysis, we studied the trends of markers of oxidative stress; endogenous non-enzymatic, enzymatic, and exogenous markers of oxidative defense; inflammatory markers, and soluble adhesion molecules in 40-to-45-year-old females across three MetS categories: controls not presenting a single MetS component, females with pre-MetS, and those with MetS. We supposed that inflammatory and oxidative status markers show similar worsening trends across the MetS categories as the components of MetS.

## 2. Materials and Methods

### 2.1. Study Design

We analyzed data obtained in the cross-sectional OBEZGEN study, which primarily aimed to evaluate the extent of genetic susceptibility in pathways leading to cardiovascular disease in unrelated 40-to-45-year-old Slovaks—Caucasians of Central European descent [57]. Study participants were recruited via general practitioners from among subjects attending preventive examinations. Exclusion criteria were any acute and chronic illnesses, pregnancy and breastfeeding, self-reported current smoking, the consumption of >50 units of alcohol/week, and the use of any bodyweight-lowering regimen. The study was conducted following the principles of the Declaration of Helsinki. The Ethics committee of the Slovak Medical University in Bratislava approved the study protocol. All subjects signed an informed consent to participate.

**Figure 1:**
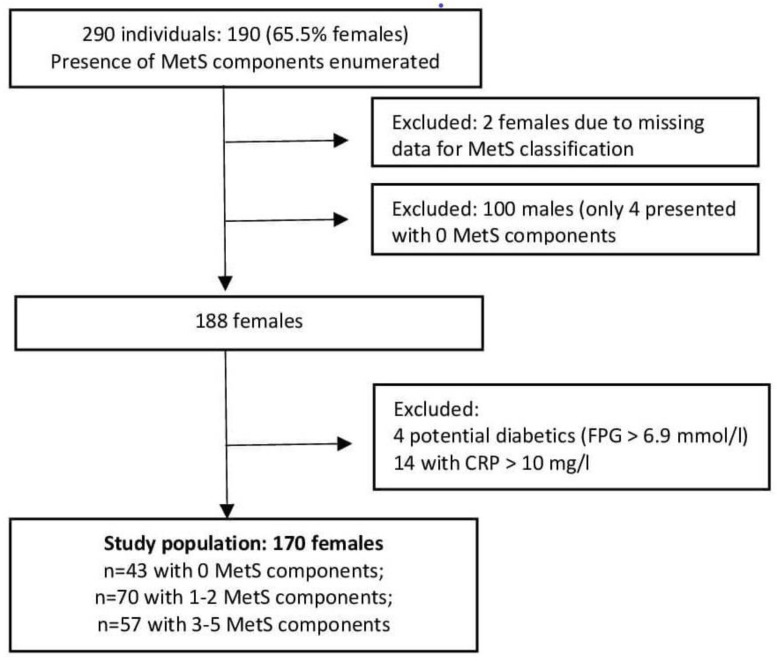
Flowchart of the study population selection.

Variables characterizing oxidative and inflammatory markers were determined in 290 participants. The presence of MetS components was enumerated according to the following criteria: central obesity—waist/height ratio (WHtR) ≥ 0.5 [58]; increased BP—systolic BP (SBP) ≥ 130 mm Hg or diastolic BP ≥ 85 mm Hg; elevated fasting plasma glucose (FPG ≥ 5.6 mmol/L); elevated TAG (≥1.7 mmol/L); low HDL-C (females: <1.3 mmol/L; males: <1.0 mmol/L) [5]. Two females with missing data for an accurate classification of MetS status were excluded. Subjects presenting with ≥3 MetS components were then considered to be suffering from MetS; pre-MetS was classified as the manifestation of one or two components; individuals not displaying a single component served as controls (CTRL). Only four males presented with zero MetS components, so this analysis was conducted only in females. After the exclusion of 18 subjects (4 potential diabetics (FPG > 6.9 mmol/L) and 14 presenting with CRP > 10 mg/L), 170 females remained for the analysis (Figure 1).

### 2.2. Measurements

Anthropometric measurements were taken by trained medical personnel according to the standard protocol. Briefly, body weight was measured using calibrated electronic scales, height was measured using an extendable stadiometer, and waist circumference was measured using a flexible tape. Body mass index (BMI) and WHtR were calculated. Blood pressure and heart rate were measured using a digital monitor (Omron Comfort, Kyoto, Japan) on the dominant arms of subjects seated for at least 5 min. The mean of the last two out of three measurements was recorded. 

Venous blood was collected from the antecubital vein after overnight fasting. Samples were coded, and analyses were performed blind. Standard blood chemistry analyses FPG, lipid profile, creatinine, uric acid, total bilirubin, and albumin, (measured using a Vitros 250 analyzer, Johnson & Johnson, Rochester, NY, USA) and blood counts (Beckman Coulter AcT 5diff hematology analyzer, Beckman Coulter, Ramsey, MN, USA) were carried out. Plasma samples were stored at −80 °C for special analyses. Fasting plasma insulin (FPI) concentrations were determined using a commercial radioimmunoassay (Immunotech, Prague, Czech Republic). C-reactive protein (CRP) was quantified nephelometrically using the IMMAGE Immunochemistry System (Beckman Coulter, Ramsey, MN, USA). Commercial ELISA sets were used according to the manufacturers’ instructions to determine IL-6, IL-10, sVAP-1, sICAM-1, and sE-selectin levels (all Bender MedSystems, Vienna, Austria), and those from R&D Systems (Minneapolis, MN, USA) were used to analyze the levels of TNF-α and sRAGE (determining all circulating sRAGE splice variants). 

Advanced-glycation-end-products-associated fluorescence of plasma (AGE-Fl) was determined fluorometrically [59] and corrected for albuminemia. The activity of semicarbazide-sensitive aminoxidase (SSAO) was measured radiometrically via liquid scintillation counting [60] and expressed as nmol of benzaldehyde formed by 1 mg of plasma protein in 1 h at 37 °C. 

All high-performance liquid chromatography (HPLC) analyses were performed using the HP 1200 LC system (Agilent Technologies, Waldbronn, Germany) equipped with a quaternary pump with an online vacuum degasser, an autosampler, a thermostatted column compartment with Peltier cooling elements, a diode array (Agilent, G1314D) or a fluorescence (Agilent G1321A) detector, and an electrically controlled internal six-port column-switching valve. A modified HPLC method was used to determine plasma levels of MDA [61]; concentrations of plasma vitamin C [62]; α-tocopherol, γ-tocopherol, β-carotene, retinol, xanthophyll, and lycopene [63]; reduced glutathione (GSH) and cysteine [64,65] were analyzed as described previously. Details regarding the columns used, mobile phases, and detection are provided in the Supplementary file.

Antioxidant enzyme activities were determined in erythrocytes lysed in distilled water after separated erythrocytes were rinsed three times with pre-cooled isotonic saline via centrifugation at 3000 rpm for 10 min at 4 °C. In diluted lysates, the activity of GPx was determined via the kinetic method according to Paglia and Valentine [66], the activity of CAT was measured spectrophotometrically using a modified method from Cavarocchi et al. [67], glutathione-S-transferase (GST) was measured using a kinetic method according to Habig et al. [68], and the activity of SOD was determined using a commercial kit (Randox Lab, Ltd., Crumlin, UK), according to the manufacturer’s instructions. Antioxidative enzyme activities were corrected for hemoglobin and quantified in lysates using the hematologic analyzer. The activity of ceruloplasmin (CPL) oxidase in plasma was measured using o-dianisidine dihydrochloride, according to the method of Schosinsky et al. [69].

### 2.3. Calculations and Classification

The concentration of tocopherol (vitamin E) was calculated as the sum of α- and γ-tocopherols, and carotenoid levels represent a sum of ß-carotene, xanthophyll, and lycopene. Tocopherol and carotenoid (=retinol + ß-carotene + lycopene + xanthophyll) concentrations were adjusted for plasma lipid levels (TAG + total cholesterol) [70,71].

The atherogenic index of plasma (AIP = log (TAG/HDL-C) [72] and the insulin sensitivity check index (QUICKI) [73] were calculated. The glomerular filtration rate (eGFR) was estimated according to the MDRD formula [74]. 

Concentrations of fasting insulin ≥ 20 μIU/mL [75], CRP > 3 mg/L [76], AIP ≥ 0.11 [72], and uric acid > 340 µmol/L were considered markers of increased cardiometabolic risk. Plasma vitamin deficiencies were classified as follows: ß-carotene < 0.37 μmol/L, retinol < 0.9 μmol/L, vitamin C < 36.1 μmol/L, and tocopherol < 12.0 μmol/L [71,77].

### 2.4. Statistical Analysis

The normality of data distribution and the equality of variances were tested using Kolmogorov–Smirnov and Levene’s tests, respectively. Skewed data were logarithmically transformed prior to the analyses. Three groups were compared using an analysis of variance (ANOVA) with a Bonferroni post-hoc test to correct for multiple comparisons. Data fitted to normal distribution are provided as means ± standard deviations (SDs); skewed data are provided as back-transformed geometric means (−1SD, +1SD). Categorical data are presented as counts (percentages) and were compared using the Chi-square test. Before the multivariate modeling, the independent variables were tested for multicollinearity. SPSS statistical software (v. 16.0 for Windows; SPSS, Chicago, IL, USA) was used with the significance set at *p* < 0.05.

Before the multivariate regression analyses, variables with high skewness and low min-to-max ratios were logarithmically transformed, all data were mean-centered, and a principal component analysis (PCA) was employed to detect outliers (Simca v.16 software, Sartorius Stedim Data Analytics AB, Umea, Sweden). The multivariate regression of independent variables on the components of MetS (dependent variables) was performed using the orthogonal projections to latent structures (OPLS) model. Variables with p_ANOVA_ < 0.1, e.g., uric acid, CRP, carotenoids/lipids, sE-selectin, leukocyte counts, IL-10, IL-6, sRAGE, total bilirubin, GSH, SSAO, cysteine, and GPx, were entered as explanatory (independent) variables. Variables with a variable of importance for the projection (VIP) value ≥ 1.00 were considered important (significant) predictors.

## 3. Results

### 3.1. General Characteristics

Out of 170 females, 43 (25.3%) did not display a single component of MetS. A total of 70 females (41.2%) presented with pre-MetS: 36 (51.4%) displayed one component, and 34 presented with two MetS components. A total of 57 females suffered from MetS: 33 (57.9%) showed three components, 20 (35.1%) presented with four components, and 4 (7.0%) presented with five components.

Measures of adiposity, BP values, and FPG rose across the categories, with significant between-group differences (Table 1). Females presenting with MetS were more insulin-resistant compared to both other groups. Except for total cholesterol concentrations, which were similar among the groups, HDL-C, TAG levels, and the AIP worsened across the groups with significant between-group differences. The estimated GFR did not differ significantly between the groups (Table 1).

The prevalence of MetS components was higher in females presenting with MetS compared with the pre-MetS group (Table 2). The prevalence of cardiometabolic risk markers increased across three groups. Central obesity, elevated SBP, and CRP were the most frequent features associated with both pre-MetS and MetS.

### 3.2. Markers of Oxidative Status

#### 3.2.1. Plasma Levels of Lipoxidation and Glycoxidation Products

Three groups displayed similar plasma MDA levels (CTRL: 0.78 ± 0.20; pre-MetS: 0.82 ± 0.27; MetS: 0.80 ± 0.19 µmol/L; *p* = 0.682). No significant difference in AGE-associated fluorescence of plasma was revealed (CTRL: 57 ± 14; pre-MetS: 55 ± 15; MetS: 52 ± 0.13 arbitrary units/g albumin; *p* = 0.160).

#### 3.2.2. Endogenous Antioxidants

Neither the activities of antioxidant enzymes in erythrocytes (Table 3) nor plasma ceruloplasmin oxidase activity differed significantly among the groups. MetS-presenting females displayed similar albumin, lower total bilirubin, and higher uric acid concentrations than their CTRL counterparts (Table 3). 

#### 3.2.3. Exogenous Antioxidants

In total, 11 (25.6%) controls, 30 (42.9%) females with pre-MetS, and 17 (29.8%) females with MetS showed vitamin C deficiencies (p_Chi_ = 0.120). The prevalence of ß-carotene deficiency reached 4.7% (n = 2), 8.6% (n = 6), and 19.3% (n = 11), respectively (p_Chi_ = 0.047). Retinol or tocopherol deficiencies were not revealed.

Vitamin C concentrations and levels of retinol, lycopene, carotenoids (retinol + ß-carotene + lycopene + xanthophyll levels), γ-tocopherol, and lipids-corrected tocopherols did not show significant between-group differences (Table 4). MetS-presenting females displayed lower plasma levels of ß-carotene and xanthophyll than the controls and lower lipids-adjusted carotenoids but higher α-tocopherol and total tocopherol levels when compared with controls and females with pre-MetS.

#### 3.2.4. Antioxidants of Mixed Origin

While the three groups displayed similar plasma concentrations of cysteine, the levels of GSH were significantly lower in females presenting with MetS vs. the controls (Table 4).

### 3.3. Markers of Inflammatory Status

#### 3.3.1. Pro- and Anti-Inflammatory Markers

Females with pre-MetS and MetS presented with higher leukocyte counts, plasma concentrations of CRP, and IL-6 than the controls, while TNF-α levels did not differ significantly (Table 5). Pre-MetS-manifesting females had higher levels of the anti-inflammatory cytokine IL-10 than the controls.

#### 3.3.2. Soluble Adhesion Molecules

Across the groups, an increasing trend was observed for sE-selectin levels, but significance was detected only between MetS-presenting and CTRL females (Table 5). Three groups displayed similar concentrations of sICAM-1 and sVAP-1. However, the SSAO activity of sVAP-1 showed a decreasing trend across the MetS groups (CTRL: 106 ± 28; pre-MetS: 96 ± 24; MetS: 93 ± 21 nmol benzaldehyde mg^−1^ (plasma protein) h^−1^; p_ANOVA_ = 0.033; CTRL vs. Mets: *p* = 0.034).

#### 3.3.3. Soluble Receptor for Advanced Glycation End Products 

Females displaying pre-MetS and MetS showed lower sRAGE levels than the controls (Table 5).

### 3.4. Multivariate Analyses

The PCA indicated that all but four scores were situated within the 95% Hotelling’s ellipse. No major outliers were detected (Supplementary Figure S1). 

Levels of multicollinearity denoted by the variance inflation factors (VIF) ranged between 1.1 and 1.3, suggesting that the regression models should not be negatively impacted. 

The multivariate regression model selected CRP, uric acid, sRAGE, carotenoids/lipids, IL-6, and leukocyte counts (VIP: 1.54 to 1.10) as significant independent predictors of the WHtR. The model described 50% of the variance in the WHtR (Table 6, Supplementary Figure S2). 

Fasting plasma glucose was predicted by carotenoids/lipids, uric acid, sRAGE, CRP, and IL-6 (VIP: 1.83 to 1.17). The model described 17% of the variance in fasting glycemia (Table 6; Supplementary Figure S3). 

Important predictors of triacylglycerolemia were carotenoids/lipids, CRP, uric acid, leukocyte counts, IL-6, sRAGE, and sE-selectin (VIP: 1.66 to 1.07). The model described 27% of the variance in TAG levels (Table 6; Supplementary Figure S4). 

SE-selectin, uric acid, IL-6, CRP, and leukocyte counts were significant predictors of HDL-C (VIP: 1.73 to 1.06) (Table 6, Supplementary Figure S5). The model explained 16% of its variance.

The OPLS model selected uric acid, sE-selectin, CRP, cysteine, IL-6, GSH, and leukocyte counts (VIP: 1.40 to 1.01) as significant predictors of SBP (Table 6, Supplementary Figure S6). The model poorly explained the variance in SBP (R^2^: 8%).

## 4. Discussion

In the first step, we looked for variable trends across the three MetS categories. Our 40-to-45-year-old females already manifested a proinflammatory phenotype at the pre-MetS stage, while an imbalance in oxidative status appeared as a feature of overt MetS. Next, we used a multiple regression model to elucidate which markers of inflammation and oxidative status are independent predictors of MetS components. Generally, higher CRP, uricemia, and IL-6 levels were associated with MetS components; leukocyte counts and sE-selectin levels showed associations with TAG, HDL-C, and SBP; and low sRAGE and carotenoid/lipid levels were associated with WHtR, FPG, and TAG. However, the impacts of individual markers varied. In addition, the percentages of the variance of individual components explained by the same independent markers of inflammation and oxidative status differed, ranging from as low as 8% in the case of SBP to 50% for the waist/height ratio. The prevalence of the component did not condition the strength of the model, e.g., the frequencies of elevated SBP and central obesity in the cohort were similar at 51% and 50%, respectively.

Defense mechanisms against oxidative stress—antioxidative enzymes and endogenous and exogenous non-enzymatic antioxidant compounds—act as radical preventive or scavenger. GPx, SOD, and catalase are the most important first-line defense antioxidants. SOD catalyzes the dismutation of two molecules of the superoxide anion to hydrogen peroxide (H_2_O_2_) and molecular oxygen (O_2_). Catalase and GPx independently convert H_2_O_2_ into water and O_2_, while GPx also breaks down lipid peroxides, utilizing GSH. Glutathione-S-transferases—phase II detoxification enzymes—also provide a powerful defense against oxidative stress by catalyzing the conjugation of GSH to electrophilic compounds. The majority of studies reported lower activities of SOD [27,28,32,48], GPx [32,48,49], and catalase [28,32,48] in the erythrocytes or whole blood of subjects with MetS than in MetS-free individuals. Similar to other studies, we revealed no significant differences between the groups’ SOD, catalase [45,48], and GPx [27,45] activities. On the other hand, in the study of Yubero-Serrano et al. [78], plasma SOD and GPx activities were lower in subjects with two MetS components than in those with four or five components. Several factors might have contributed to contradictory results: the study groups differed by age (even by decades in mean), the majority of the studies did not consider sex as a biological variable, the sources of biological material used for the analyses (erythrocytes, whole blood, and plasma) differed, and lifestyle factors, including the dietary intake of micronutrients essential as cofactors of antioxidative enzymes, might have been dissimilar. Moreover, except for redox status, antioxidative enzyme activities are regulated by genetic factors. To our knowledge, data on the activity of GST in MetS patients are unavailable. Interestingly, we observed no significant differences in the activities of GSH-dependent enzymes, despite the fact that the concentration of GSH—a major intracellular non-protein thiol involved in antioxidant defense—declined across the MetS groups. Data on GSH levels in subjects with MetS are controversial: both lower [48,79] or similar [46] levels compared with MetS-free subjects were reported. GSH declined across the MetS categories despite the levels of cysteine—the rate-limiting precursor for GSH (γ-glutamyl-cysteinyl-glycine) synthesis and a potent antioxidant—remaining unaltered. Data on cysteinemia in subjects with MetS are equivocal: lower levels in MetS [79] or even a 10-fold increase in subjects already presenting with one MetS component [47] were reported. Lower concentrations of GSH in cases of unaltered cysteinemia and GSH-associated enzyme activities suggest either a decreased activity of GSH-reductase or the consumption of GSH via conjugation, hydrolysis, or other regulatory pathways [80]. Ceruloplasmin acts, among others, as a free radical scavenger and an acute phase reactant. While some studies reported that subjects with MetS displayed higher ceruloplasmin levels than their MetS-free peers [50,51], in line with another study [52], we revealed no significant differences. Similar activities of antioxidant enzymes across the MetS categories in our study suggest that this defense system copes with stress conditions, even under a mild decline in GSH levels. 

Plasma albumin, bilirubin, and uric acid represent non-enzymatic antioxidants synthesized in the liver. Due to its high concentrations in plasma, multiple ligand-binding capacities, and free-radical-trapping properties, albumin is the main extracellular molecule responsible for maintaining the plasma redox state. Similar to other studies [27,29], we revealed no significant difference in albuminemia among the MetS groups, although Suriyaprom et al. [28] reported lower levels in subjects with MetS. Bilirubin, the final product of heme catabolism, is the most potent endogenous antioxidant protecting lipids. By inhibiting TNF-α-induced adhesion molecules, it exerts anti-inflammatory effects, and via binding to the transcription factor peroxisome proliferator-activated receptor-α, it activates downstream pathways which improve, among others, insulin resistance, hypertension, and obesity [81,82,83]. In contrast to studies reporting similar total bilirubin concentrations in subjects with and without MetS [27,44,46], our females suffering from MetS displayed lower levels than the controls. Mohorko et al. [47] observed lower bilirubinemia already in subjects with ≥2 MetS components compared to those with ≤1. A decline in bilirubin concentrations within the reference range is of clinical importance as at levels <9 µmol/L, the endocrine effect of bilirubin decreases. Low bilirubinemia is associated with obesity, non-inflammatory fatty liver, and cardiovascular diseases and increases the odds of developing MetS [82]. In humans, uric acid is the bioactive end-product of purine metabolism. Except for endogenous production, plasma uric acid levels depend on the dietary intake of purines and fructose and the renal and intestinal excretion of urates. Uric acid has a bimodal role in maintaining oxidative balance: extracellularly, it acts as an antioxidant, while intracellular uric acid acts as a pro-oxidant either directly, by generating ROS, or indirectly, via stimulating proinflammatory biomarkers [84,85]. Our data confirm the former observations of higher uricemia in subjects with MetS than in the controls [27,43,44,45,46]. Experimental and clinical studies suggest that uric acid is a true modifying and possibly causal factor for essential hypertension, insulin resistance, atherogenic dyslipidemia, and decline in renal function [86,87,88]. According to Virdis et al. [89], uric acid levels increasing the risk of total and cardiovascular mortality are significantly lower than those used to define hyperuricemia in clinical practice. On the other hand, our former study showed that hyperuricemia is associated with cardiometabolic risk indicators only in overweight/obese but not in lean adolescents [90]. Thus, it is questionable whether higher MetS-associated uricemia should be interpreted as a beneficial effect of activated adaptive mechanisms to cope with increased oxidative stress or as an indicator of increased cardiometabolic risk.

Vitamin C (ascorbate) acts as a potent scavenger and contributes to the resolution of the inflammatory process [91]. In our females, ascorbate levels were similar across the MetS categories. A recent meta-analysis of observational studies showed that subjects with MetS generally present with lower vitamin C levels than MetS-free controls [92]. Vitamin C and E are co-nutrients, as vitamin C regenerates the tocopheryl from its oxidized form. Among tocopherol derivatives, α-tocopherol has the highest bioavailability. Although our females with MetS displayed the highest α-tocopherol levels among the MetS categories, and an increasing tendency was observed for γ-tocopherol, if corrected for lipidemia, three groups displayed similar tocopherolemia. The latest meta-analysis deduced that the current evidence is still insufficient to conclude a relationship between the level of circulating vitamin E and MetS [93]. Dietary carotenoids are either phytochemicals (such as ß-carotene and oxidative derivatives of carotenes—xanthophylls or lycopene) or come from animal sources (e.g., retinol). Although the plasma levels of carotenoids are of an order of magnitude lower than those of tocopherols, the resultant free-radical scavenging and antioxidant capacities of carotenoids and tocopherols are of comparable magnitudes [83]. Except for oxidative stress, carotenoids positively modulate inflammation, especially in pathways related to the nuclear factor κ-B. Adults with MetS presented either with lower [77] or higher plasma levels of retinol [70,94,95,96], while our females displayed similar levels across the MetS categories. On the other hand, carotenoids of plant origin—ß-carotene and xanthophyll but not lycopene—were lower in females suffering from MetS. Our data on plasma ß-carotene and total carotenoid levels align with other studies [70,95,96]. Whether lower levels of ß-carotene and xanthophyll reflected low dietary intake remains unclear, as we have no data on the diets of our females. In the multivariate regression, lipids-corrected carotenoids were the only dietary compounds significantly associated with MetS components. Regarding lycopene, higher [95], lower [70], or, as in our study, similar [96] plasma levels in MetS-presenting and MetS-free individuals were reported. A recent review concludes that studies generally support protective relations between lycopene and MetS, but different components appear to be influenced rather than demonstrating consistent improvement in a single component [97]. A comparison of the data on the plasma levels of antioxidant vitamins between different studies in subjects with MetS is cumbersome. Except for dietary intake, which generally reflects seasonal variability in the supply of fresh fruits and vegetables, geographical location, age, sex, physical activity, and other determinants play a role. 

Malondialdehyde—a widely used biomarker for lipid peroxidation—is an end product of the decomposition of arachidonic acid and larger polyunsaturated fatty acids [98]. Data on the concentrations of MDA in subjects with MetS differ: higher plasma levels than in subjects without MetS [10,43,44,48,49], as well as similar concentrations [27,32]—as observed in our study—were reported. Unaltered MDA levels may reflect its effective detoxification by glutathione [80], the fact that MDA forms advanced lipoxidation end products, or even that it might be converted into methylglyoxal [98]. Methylglyoxal is effectively detoxified by glyoxalase I, for which glutathione acts as a cofactor [99]. However, a minor fraction of methylglyoxal escaping the glyoxalase system may react with proteins to form AGEs. Whether lower plasma GSH levels, as observed in our study, might boost the formation of methylglyoxal-derived AGEs remains unclear. Major methylglyoxal-derived AGEs have no intrinsic fluorescence and thus could not be captured via the determination of AGE-associated fluorescence of plasma. 

AGE is a collective name for different irreversibly modified proteins formed after exposure to hyperglycemia or oxidative stress [100,101]. The toxicity of AGE-modified proteins lies in their altered structure and function. Furthermore, via interaction with their receptor RAGE, AGEs elicit downstream pathways, resulting in the generation of ROS and proliferative, inflammatory, and thrombogenic responses playing a role in the development and progression of numerous diseases and disorders associated with MetS [102,103,104]. We did not reveal significant differences in AGE-Fl/Alb across the MetS categories, in line with our former study in adults [53]. Reports on chemically-defined non-fluorescent AGE—N^ε^-(carboxymethyl)lysine—in subjects with MetS mention either higher levels than in MetS-free ones [105] or describe its inverse association with the number of MetS components [53]. Different analytical approaches, cohort characteristics, diets (dietary AGEs are partially absorbed into circulation), renal function (the kidney plays a key role in the disposal of low-molecular-weight AGEs), and different prevalences of obesity (which is associated with low levels of circulating AGE) might account for the discrepant results [106,107,108].

Soluble RAGEs are formed either via the proteolytic cleavage of a cell surface RAGE or via the alternative splicing of RAGE pre-mRNA. As these isoforms carry the ligand-binding domain, sRAGE may act as a decoy receptor. By blocking the interaction of RAGE ligands with the cell surface RAGE, sRAGEs can reduce oxidative stress and inflammatory reactions. We confirmed the former data that sRAGE levels are lower in subjects with MetS than in the controls and decline with the number of MetS components manifested [53,54,56,104]. Except for AGEs, other proinflammatory molecules, such as S100/calgranulins and amphoterin, serve as RAGE ligands [109]; thus, inflammation may be a key factor linking the RAGE system with MetS. This fits our finding that sRAGE was a significant predictor for the MetS components in multivariate analyses.

MetS is a state of sterile inflammation characterized by increased total leukocyte numbers, the activation of inflammatory signaling pathways, abnormal cytokine production, and an increased acute-phase response [110]. In our study, even females with pre-MetS displayed higher leukocyte counts than the controls. Other [10,27,28] studies, but not all [29,30] studies, also reported higher leukocyte numbers in subjects with MetS. 

Visceral adipose tissue is metabolically active and engaged in cross-talk between the organs, contributing to the development of insulin resistance and atherosclerosis [14,17]. Visceral adipocytes per se produce adipokines and proinflammatory cytokines, such as IL-6 and TNF-α. In obesity, macrophages and leukocytes attracted by apoptotic and necrotic hypertrophic visceral adipocytes further contribute to the production of ROS and excrete cytokines into portal blood directly flowing into the liver. Here, they induce inflammation and oxidative stress response, contributing to the development of insulin resistance and atherogenic dyslipidemia. The release of the inflammatory cytokine IL-6 is stimulated, among others, by oxidative stress and obesity. Under these conditions, IL-6 propagates the inflammatory response by boosting the synthesis of acute-phase reactants, such as CRP, in the liver [14]. In line with this, our females with pre-MetS and MetS displayed higher IL-6 and CRP levels than the controls. Clinical studies concurrently determining IL-6 and CRP also reported higher levels of these inflammatory markers in subjects with MetS than in those without [31,32,33,34,50,111,112]. Another inflammatory cytokine produced primarily by macrophages—TNF-α—induces the production of acute-phase reactants in the liver and insulin resistance [14]. Surprisingly, and in contrast with other authors reporting higher TNF-α levels in MetS subjects than in those without [10,28,30,31,33,34,50], we observed similar levels of TNF-α across the MetS categories. IL-10 is an anti-inflammatory cytokine produced mainly by macrophages. It beneficially affects insulin signaling via restoring its dysregulation due to abnormal levels of the proinflammatory cytokines IL-6 and TNF-α and by inhibiting oxidative stress [113]. Data on IL-10 levels in subjects with MetS are also equivocal: lower [37,112], similar [28], or higher levels [33] than in MetS-free subjects have been reported. We observed an increasing trend across the MetS categories, with significantly higher levels in pre-MetS females compared with the controls. Our results indicate that inflammatory markers (leukocyte counts, CRP, IL-6, and sRAGE) are important predictors for MetS components. Discrepant results on the markers of inflammatory status in MetS might reflect different prevalences of MetS components in individual cohorts and different associations of MetS components with single inflammatory markers. 

Endothelial dysfunction is an early pathogenetic event in MetS. Cellular adhesion molecules (CAMs) govern the rolling of leukocytes, their adhesion to endothelial cells, and their extravasation into the tissues. Proinflammatory cytokines and acute-phase proteins release soluble CAMs. SCAMs are considered reliable and sensitive biomarkers for endothelial activation associated with proinflammatory phenotype [114]. VAP-1 is a unique adhesion molecule, possessing the enzyme activity of SSAO, which converts primary amines into corresponding aldehydes (e.g., aminoacetone into methylglyoxal), concurrently producing ammonia and H_2_O_2_ [115]. On adipocytes, VAP-1 has insulin-like effects. Although patients with diabetes present with higher sVAP-1 levels, this pathway probably plays only a minor role under physiological conditions [115]. The presence of MetS did not associate with altered sVAP-1 levels, corresponding with our former results in adolescents [42]. While sE-selectin comes exclusively from activated endothelium and is considered conclusive evidence of endothelial activation [114], sICAM-1 is a product of many cells except for endothelial cells, mainly of leukocytes [116]. Among the three soluble CAMs studied, only E-selectin showed an increasing trend across the MetS categories, with significance between MetS and control females. Studies simultaneously determining sE-selectin and sICAM-1 levels reported either higher levels of both sCAMs in subjects with MetS than in the controls [37,39] or, similar to our observation, only higher levels of sE-selectin [40,41]. In subjects with MetS, sE-selectin levels correlated directly with measures of central obesity, blood pressure, insulinemia, and TAG levels and inversely with HDL-C [39,40,41]. In our multivariate analyses, sE-selectin was similarly and significantly associated with TAG, SBP, and HDL-C levels. Endothelial activation precedes endothelial damage related to the production of ROS and inflammation, eventually stimulating the development of atherosclerotic lesions [114]. On the other hand, the shedding of E-selectin reduces its density on endothelium, thus resulting in decreased leukocyte adhesion to the vascular wall, concurrently generating a decoy ligand that competes with membrane-bound E-selectin for the binding of leukocytes [117]. Thus, longitudinal clinical and animal model studies are needed to clarify the dynamics, interplay, and exact role of sCAMs in the development of MetS.

Our study is limited by the fact that we present an analysis of a cross-sectional survey in females; thus, our data can neither be extrapolated to other populations nor be interpreted other than with respect to associations. We have no data on dietary intake and other lifestyle factors that could affect oxidative and inflammatory status. On the other hand, our strength is that we report on a reasonably large panel of inflammatory and oxidative stress markers in pre-menopausal females of similar ages. Our probands were sampled exclusively during the spring months to minimize seasonal variations in antioxidant levels. Except for describing the trends in oxidative and inflammatory markers across three categories (healthy, presenting with pre-MetS, or MetS), we show that associations of these markers with single components of MetS differ. 

## 5. Conclusions

Our results showed that in 40-to-45-year-old females, the associations of the same markers of oxidative status and inflammation with individual risk components differ considerably. The burden of inflammatory and oxidative stress in patients with MetS is not uniform—it reflects the composition and severity of manifested components rather than their association with MetS, which was diagnosed as the presence of any three of the five components. Among the four markers consistently appearing as predictors of single components, leukocyte counts, uric acid, and CRP are routinely available in clinical practice. A question arises whether awareness of their elevation within their reference ranges could aid in the early detection and management of individuals who are at risk for developing MetS or who already exhibit some clustered components of MetS.

## Figures and Tables

**Table 1:** General characteristics.

	CTRL (n = 43)	Pre-MetS (n = 70)	MetS (n = 57)	*p*
Age, years	41.7 ± 1.4	42.2 ± 1.5	42.6 ± 1.4 *	0.053
Waist circumference, cm	73.7 ± 5.9	83.8 ± 10.8 ***	89.1 ± 8.9 ***^,+++^	**<0.001**
Body mass index, kg/m^2^	22.1 ± 2.2	26.6 ± 4.6 ***	30.8 ± 4.7 ***^,+++^	**<0.001**
Waist/height	0.44 ± 0.03	0.51 ± 0.06 ***	0.57 ± 0.06 ***^,+++^	**<0.001**
Systolic BP, mm Hg	116 ± 7	132 ± 14 ***	136 ± 14 ***	**<0.001**
Diastolic BP, mm Hg	74 ± 6	83 ± 9 ***	89 ± 9 ***^,++^	**<0.001**
FPG, mmol/L	5.0 ± 0.3	5.2 ± 0.4 *	5.6 ± 0.5 ***^,+++^	**<0.001**
FPI, µIU/mL	7.9 ± 3.5	9.5 ± 7.2	13.9 ± 8.6 ***^,++^	**<0.001**
QUICKI	0.356 ± 0.023	0.349 ± 0.028	0.324 ± 0.021 ***^,+++^	**<0.001**
Total cholesterol, mmol/L	4.98 ± 0.85	5.57 ± 0.88	5.35 ± 0.89	0.181
HDL-C, mmol/L	1.76 ± 0.39	1.52 ± 0.32 ***	1.22 ± 0.26 ***^,++^	**<0.001**
Triacylglycerols, mmol/L	0.85 ± 0.29	1.14 ± 0.46 *	1.84 ± 0.69 ***^,+++^	**<0.001**
AIP	−0.33 ± 0.17	−0.14 ± 0.20 ***	0.16 ± 0.20 ***^,+++^	**<0.001**
eGFR, mL/min/1.73 m^2^	1.5 ± 0.3	1.6 ± 0.3	1.6 ± 0.3	0.363

CTRL, zero components of metabolic syndrome (MetS); Pre-MetS, 1 or 2 components of MetS; MetS, ≥3 components of MetS; BP, blood pressure; FPG, fasting plasma glucose; FPI, fasting plasma insulin; IU, international units; QUICKI, quantitative insulin sensitivity check index; HDL-C, high-density lipoprotein cholesterol; AIP, atherogenic index of plasma; eGFR, estimated glomerular filtration rate. Data are provided as means ± SDs. Three groups were compared using an analysis of variance (ANOVA) with a post-hoc Bonferroni test to correct for multiple comparisons. *: *p* < 0.05 vs. CTRL; ***: *p* < 0.001 vs. CTRL; ^++^: *p* < 0.01 vs. Pre-MetS; ^+++^: *p* < 0.001 vs. Pre-MetS; *p* given in bold is significant.

**Table 2:** The prevalence of cardiometabolic risk factors and markers.

	CTRL (n = 43)	Pre-MetS (n = 70)	MetS (n = 57)	*p*
Waist/height ≥ 0.5	NA	32 (45.7)	53 (93.0)	**<0.001**
Systolic BP ≥ 130 mm Hg	NA	40 (57.1)	46 (80.7)	**0.007**
FPG ≥ 5.6 mmol/L	NA	13 (18.6)	34 (59.6)	**<0.001**
HDL-C < 1.3 mmol/L	NA	14 (20.0)	36 (63.2)	**<0.001**
Triacylglycerols ≥ 1.7 mmol/L	NA	5 (7.1)	30 (52.6)	**<0.001**
BMI > 29.9. kg/m^2^	0	14 (20.0)	33 (57.9)	**<0.001**
Diastolic BP ≥ 85 mm Hg	3 (7.0)	29 (41.4)	40 (70.2)	**<0.001**
FPI ≥ 20 µIU/mL	0	2 (2.9)	8 (14.0)	**0.005**
AIP ≥ 0.11	0	8 (11.4)	35 (61.4)	**<0.001**
Uric acid > 340 µmol/L	0	1 (1.4)	5 (8.8)	**0.029**
C-reactive protein > 3 mg/L	21 (48.8)	47 (67.1)	51 (89.5)	**<0.001**

CTRL, zero components of metabolic syndrome (MetS); Pre-MetS, 1 or 2 components of MetS; MetS, ≥3 components of MetS; BP, blood pressure; FPG, fasting plasma glucose; HDL-C, high-density lipoprotein cholesterol; BMI, body mass index; FPI, fasting plasma insulin; IU, international units; AIP, atherogenic index of plasma. Data are provided as counts (percentages). The groups were compared using the Chi-square test; *p* given in bold is significant.

**Table 3:** Oxidative status: endogenous antioxidants.

	CTRL (n = 43)	Pre-MetS (n = 70)	MetS (n = 57)	*p*
Superoxide dismutase, U/g Hb	1574 ± 452	1632 ± 386	1652 ± 391	0.628
Catalase, kU/g Hb	11.8 ± 4.7	11.5 ± 4.4	11.4 ± 5.4	0.929
Glutathione peroxidase, U/g Hb	10.0 ± 3.8	9.4 ± 4.3	11.0 ± 3.6	0.082
Glutathione-S-transferase, U/g Hb	44.2 ± 8.7	44.1 ± 9.7	44.2 ± 9.4	0.998
Ceruloplasmin oxidase, U/L	89 ± 44	103 ± 35	97 ± 30	0.145
Albumin, g/L	44.9 ± 3.3	45.1 ± 4.2	44.3 ± 3.5	0.529
Total bilirubin, µmol/L	10.0 ± 5.9	9.4 ± 4.7	7.7 ± 3.8 *	**0.033**
Uric acid, µmol/L	214 ± 54	239 ± 49	262 ± 64 ***	**<0.001**

CTRL, zero components of metabolic syndrome (MetS); Pre-MetS, 1 or 2 components of MetS; MetS, ≥3 components of MetS; Hb, hemoglobin. Data are provided as means ± SDs; groups were compared using an analysis of variance (ANOVA) with a post-hoc Bonferroni test to correct for multiple comparisons. *: *p* < 0.05 vs. CTRL; ***: *p* < 0.001 vs. CTRL; *p* given in bold is significant.

**Table 4:** Oxidative status: antioxidants of exogenous or mixed origin.

	CTRL (n = 43)	Pre-MetS (n = 70)	MetS (n = 57)	*p*
Vitamin C, µmol/L	49.3 ± 19.4	44.8 ± 21.0	46.8 ± 20.3	0.528
Retinol, µmol/L	2.32 ± 0.67	2.52 ± 0.81	2.55 ± 0.81	0.286
ß-carotene, µmol/L	1.20 ± 0.73	1.01 ± 0.60	0.86 ± 0.59 *	**0.032**
Lycopene, µmol/L	0.50 ± 0.28	0.43 ± 0.20	0.47 ± 0.24	0.353
Xanthophyll, µmol/L	0.30 ± 0.15	0.25 ± 0.16	0.22 ± 0.13 *	**0.022**
Carotenoids, µmol/L	4.32 ± 1.21	4.22 ± 1.06	4.10 ± 1.22	0.630
Carotenoids/lipids, µmol/mmol	0.75 ± 0.21	0.68 ± 0.23	0.59 ± 0.19 ***^,+^	**<0.001**
α-tocopherol, µmol/L	26.6 ± 7.2	27.4 ± 6.3	31.3 ± 7.8 **^,++^	**0.001**
γ-tocopherol, µmol/L	1.09 ± 0.59	1.14 ± 0.55	1.33 ± 0.65	0.088
Tocopherols, µmol/L	27.7 ± 7.5	28.6 ± 6.4	32.6 ± 8.0 **^,++^	**0.001**
Tocopherols/lipids, µmol/mmol	4.8 ± 1.2	4.6 ± 1.0	4.6 ± 1.0	0.612
Cysteine, µmol/L	225 ± 40	220 ± 40	207 ± 42	0.081
GSH, µmol/L	9.1 ± 2.2	8.3 ± 2.6	7.9 ± 2.4 *	**0.041**

CTRL, zero components of metabolic syndrome (MetS); Pre-MetS, 1 or 2 components of MetS; MetS, ≥3 components of MetS; carotenoids, retinol + ß-carotene + lycopene + xanthophyll; lipids; plasma triacylglycerols + total cholesterol concentrations; GSH, reduced glutathione. Data are provided as means ± SDs; three groups were compared using an analysis of variance (ANOVA) with a post-hoc Bonferroni test to correct for multiple comparisons. *: *p* < 0.05 vs. CTRL; **: *p* < 0.01 vs. CTRL; ***: *p* < 0.001 vs. CTRL; ^+^: *p* < 0.05 vs. Pre-MetS; ^++^: *p* < 0.01 vs. Pre-MetS; *p* given in bold is significant.

**Table 5:** Markers of inflammatory status.

	CTRL (n = 43)	Pre-MetS (n = 70)	MetS (n = 57)	*p*
Leukocytes, 10^9^/L	6.0 ± 1.4	6.9 ± 1.6 *	7.1 ± 1.7 **	**0.002**
C-reactive protein, mg/L	3.2 ± 1.5	4.7 ± 2.8 **	5.4 ± 2.1 ***	**<0.001**
Interleukine-6, pg/mL	0.2 (0.1; 0.6)	0.4 (0.1; 1.6) *	0.4 (0.1; 1.7) *	**0.014**
TNF-α, pg/mL	5.2 ± 2.2	5.0 ± 2.6	5.2 ± 2.7	0.927
Interleukine-10, pg/mL	2.0 (0.8; 4.9)	3.3 (1.5; 7.5) **	2.6 (1.2; 5.9)	**0.006**
sE-selectin, ng/mL	47.2 ± 22.8	57.2 ± 30.2	66.3 ± 32.1 **	**0.006**
sICAM-1, ng/mL	152 ± 26	152 ± 27	150 ± 24	0.903
sVAP-1, ng/mL	262 ± 77	260 ± 80	257 ± 75	0.953
sRAGE, pg/mL	1683 ± 573	1412 ± 402 *	1373 ± 428 *	**0.010**

CTRL, zero components of metabolic syndrome (MetS); Pre-MetS, 1 or 2 components of MetS; MetS, ≥3 components of MetS; TNF-α, tumor necrosis factor-α; s, soluble; ICAM, intracellular adhesion molecule; VAP, vascular adhesion molecule; RAGE, the receptor for advanced glycation end products. Normally distributed data are provided as means ± SDs; data not fitted to a normal distribution are provided as back-transformed geometric means (−1SD; +1SD); three groups were compared using an analysis of variance (ANOVA) with a post-hoc Bonferroni test to correct for multiple comparisons. *: *p* < 0.05 vs. CTRL; **: *p* < 0.01 vs. CTRL; ***: *p* < 0.001 vs. CTRL; *p* provided in bold is significant.

**Table 6:** Multivariate regression of selected inflammatory and oxidative status markers (independent variables) on waist/height ratio, fasting plasma glucose, triacylglycerols, high-density lipoprotein cholesterol, and systolic blood pressure (dependent variables), using the orthogonal projections to latent structures model.

	WHtR	FPG	TAG	HDL-C	SBP
C-reactive protein	**1.54**	**1.20**	**1.38**	**1.23**	**1.26**
Uric acid	**1.35**	**1.46**	**1.26**	**1.34**	**1.40**
sRAGE	**1.28**	**1.46**	**1.09**	0.92	0.59
Carotenoids/lipids	**1.28**	**1.83**	**1.66**	0.87	0.88
Interleukine-6	**1.13**	**1.17**	**1.13**	**1.28**	**1.09**
Leukocytes	**1.10**	0.95	**1.26**	**1.06**	**1.01**
E-selectin	0.86	0.85	**1.07**	**1.73**	**1.34**
Interleukine-10	0.80	0.78	0.59	0.46	0.70
Glutathione peroxidase	0.64	0.33	0.42	0.75	0.96
GSH	0.63	0.93	0.67	0.64	**1.09**
Cysteine	0.62	0.64	0.44	0.43	**1.20**
SSAO	0.55	0.65	0.49	0.72	0.28
Total bilirubin	0.45	0.75	0.35	0.66	0.47
R^2^	0.50	0.17	0.27	0.16	0.08

WHtR, waist-to-height ratio; FPG, fasting plasma glucose; TAG, triacylglycerols; HDL-C, high-density lipoprotein cholesterol; SBP, systolic blood pressure; s, soluble; RAGE, the receptor for advanced glycation end products; carotenoids, retinol + ß-carotene + xanthophyll + lycopene; lipids, total cholesterol + triacylglycerols; GSH, reduced glutathione; SSAO, semicarbazide-sensitive amine oxidase. Variables with a variable of importance for the projection values ≥ 1.00 were considered important (significant) contributors (provided in bold).

## Data Availability

Data are available upon request from the corresponding author.

## Supplementary Materials

The following supporting information can be downloaded at: https://www.mdpi.com/article/10.3390/antiox12061221/s1, Figure S1: Score plot—principal component analysis; Supplementary Figure S2: Scatter plot: multivariate regression of independent variables—markers of oxidative status and inflammation on waist/height ratio using the orthogonal projection to latent structures model; Supplementary Figure S3: Loading scatter plot: multivariate regression of independent variables—markers of oxidative status and inflammation on fasting plasma glucose using the orthogonal projection to latent structures model; Supplementary Figure S4: Loading scatter plot—multivariate regression of independent variables—markers of oxidative status and inflammation on triacylglycerols using the orthogonal projection to latent structures model; Supplementary Figure S5: Loading scatter plot—multivariate regression of independent variables—markers of oxidative status and inflammation on high-density lipoprotein cholesterol using the orthogonal projection to latent structures model; Supplementary Figure S6: Loading scatter plot—multivariate regression of independent variables—markers of oxidative status and inflammation on systolic blood pressure using the orthogonal projection to latent structures model.
