# Supplementary material for: Association of Inflammatory and Oxidative Status Markers with Metabolic Syndrome and Its Components in 40-to-45-Year-Old Females: A Cross-Sectional Study
Source: Antioxidants (Basel). 2023 Jun 5;12(6):1221. doi: 10.3390/antiox12061221 (PMC10295263; doi:10.3390/antiox12061221)
Supplement: Supplementary file 1 [file antioxidants-12-01221-s001.zip › antioxidants-2398408-supplementary.pdf]

Supplementary Materials

Association of Inflammatory and Oxidative Status Markers with Metabolic Syndrome and Its Components in 40-To-45-Year-Old Females: A Cross-Sectional Study

Katarína Šebeková [1,*], Marta Staruchová [2], Csilla Mišľanová [3], Aurélia Líšková [4], Mira Horváthová [4], Jana Tulinská [4], Miroslava Lehotská Mikušová [4], Michaela Szabová [4], Radana Gurecká [1,5], Ivana Koborová [1], Melinda Csongová [1], Tamás Tábi [6], Éva Szökö [6] and Katarína Volkovová [2]

[1] Institute of Molecular Biomedicine, Medical Faculty, Comenius University in Bratislava, 83303 Bratislava, Slovakia; radana.kollarova@gmail.com (R.G.); koborova@gmail.com (I.K.); melinda.csongova@gmail.com (M.C.)
[2] Institute of Biology, Medical Faculty, Slovak Medical University in Bratislava, 83303 Bratislava, Slovakia; marta.staruchova@szu.sk (M.S.); katarina.volkovova@szu.sk (K.V.)
[3] Institute of Nutrition, Faculty of Nursing and Medical Professional Studies, Slovak Medical University in Bratislava, 83303 Bratislava, Slovakia; csilla.mislanova@szu.sk
[4] Department of Immunology and Immunotoxicology, Slovak Medical University in Bratislava, 83303 Bratislava, Slovakia; aurelia.liskova@szu.sk (A.L.); mira.horvathova@szu.sk (M.H.); jana.tulinska@szu.sk (J.T.); miroslava.mikusova@szu.sk (M.L.M.); michaela.szabova@szu.sk (M.S.)
[5] Institute of Medical Physics, Biophysics, Informatics and Telemedicine, Faculty of Medicine, Comenius University in Bratislava, 83303 Bratislava, Slovakia
[6] Department of Pharmacodynamics, Faculty of Pharmacy, Semmelweis University, 1085 Budapest, Hungary; tabi.tamas@pharma.semmelweis-uni.hu (T.T.); eva.szoko@pharma.semmelweis-uni.hu (É.S.)
* Correspondence: katarina.sebekova@imbm.sk

Supplementary file: Description of the HPLC methods

All high-performance liquid chromatography (HPLC) analyses were performed using the HP 1200 LC system (Agilent Technologies, Waldbronn, Germany) equipped with a quarternary pump with an on-line vacuum degasser, an autosampler, a thermostatted column compartment with Peltier cooling elements, a diode-array or a fluorescence detector, and an electrically controlled internal six-port column switching valve.

Malondialdehyde
Analytical column: LiChrospher® RP-18 (125x4 mm, I.D., 5 µm, Merck, Darmstadt, Germany)
Precolumn: LiChrospher® RP-18 (10x4 mm, I.D., 5 µm, Merck, Darmstadt, Germany)
Mobile phase (isocratic elution): 50 mmol/L phosphate buffer, pH 6.8 : methanol (60:40, v/v)
Flow rate: 1.5 mL/min
Injection volume: 50 µL
Fluorescence detection: λ_{exc} = 532 nm / λ_{em} = 553 nm.

Richard, M. J.; Guiraud, P.; Meo, J.; Favier, A., High-performance liquid chromatographic separation of malondialdehyde-thiobarbituric acid adduct in biological materials (plasma and human cells) using a commercially available reagent. *J Chromatogr* **1992**, *577*, 9-18

Vitamin C
Analytical column: LiChrospher RP 18 reversed-phase column (250x4 mm, I.D., 5 µm, Merck, Darmstadt, Germany
Precolumn: LiChrospher RP 18 (10x4 mm, I.D, 5 µm, Merck, Darmstadt, Germany)
Mobile phase: 3.7 mmol/L potassium dihydrogen phosphate, pH 4.4
Flow rate: 0.8 mL/min
Injection volume: 25 µL
UV detection: 245 nm.

Cerhata, D.; Bauerová, A.; Ginter, E., [Determination of ascorbic acid in blood serum using high-performance liquid chromatography and its correlation with spectrophotometric (colorimetric) determination]. *Ceska Slov Farm* **1994**, *43*, 166-8

α-tocopherol, γ-tocopherol, β-carotene, retinol, xanthophyll, and lycopene

Analytical column: Nucleosil 120-C18 (250x4.6 mm, I.D., 5 μm, Merck, Darmstadt, Germany)
Precolumn: Nucleosil 120-C18 (10x4 mm, I.D, 5 μm, Merck, Darmstadt, Germany)
Mobile phase: acetonitrile/tetrahydrofurane/methanol+butylhydroxytoluene/1 % ammonium acetate (67.4:22:6.8:3.8, v/v/v/v)
Flow rate: 1.2 mL/min
Injection volume: 20 μL
Column temperature: 29 °C.
Detection: UV (λ= 450 nm) and fluorescence detectors connected in series. Fluorescence detection was programmed as follows: 0-4 min 330/470 ($\lambda_{exc}/\lambda_{em}$); 4.1-10 min 298/328 ($\lambda_{exc}/\lambda_{em}$)

Hess, D.; Keller, H. E.; Oberlin, B.; Bonfanti, R.; Schüep, W., Simultaneous determination of retinol, tocopherols, carotenes and lycopene in plasma by means of high-performance liquid chromatography on reversed phase. *Int J Vitam Nutr Res* **1991,** *61*, 232-8.

Reduced glutathione (GSH) and cysteine

Analytical column: Purospher STAR C18 (250x4.6 mm, I.D., 5 μm, Merck, Darmstadt, Germany)
Precolumn: Purospher® STAR RP-18e (10x4mm, I.D., 5 μm, Merck, Darmstadt, Germany)
Mobile phase: 50.0 mmol/L sodium dihydrogenphosphate, 1.0 mmol/L ion-pairing reagent n-octylsulphonic acid (OSA), 6% acetonitrile (v/v/v); pH 2.60 with 85% (v/v) phosphoric acid
Flow rate: 0.65 mL/min
Injection volume: 20 μL
Detection: Electrochemical detection using a coulometric detector (Coulochem II, ESA, Inc., Chelmsford, MA, USA) equipped with an analytical cell (model 5010A) and a guard cell (model 5020). The electrode potentials for the E1, E2 of analytical cell and guard cell were set at +650 mV, +900 mV and +1400 mV, respectively. Reference electrode: Ag/AgCl.

Houze, P.; Gamra, S.; Madelaine, I.; Bousquet, B.; Gourmel, B., Simultaneous determination of total plasma glutathione, homocysteine, cysteinylglycine, and methionine by high-performance liquid chromatography with electrochemical detection. *J Clin Lab Anal* **2001,** *15*, 144-53; Melnyk, S.; Pogribna, M.; Pogribny, I.; Hine, R. J.; James, S. J., A new HPLC method for the simultaneous determination of oxidized and reduced plasma aminothiols using coulometric electrochemical detection. *J Nutr Biochem* **1999,** *10*, 490-7.

Supplementary Figure S1. Score plot – principal component analysis

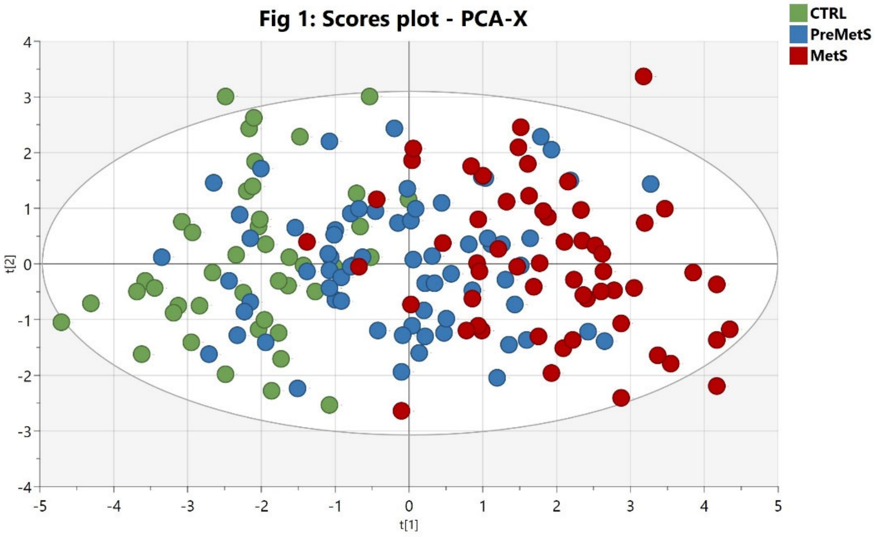

CTRL: females not presenting a single component of metabolic syndrome; PreMetS: females presenting with 1 or 2 components; MetS: females with overt metabolic syndrome (3-to-5 components present)

Supplementary Figure S2. Scatter plot – multivariate regression of independent variables - markers of oxidative status and inflammation on waist/height ratio using the orthogonal projection to latent structures model

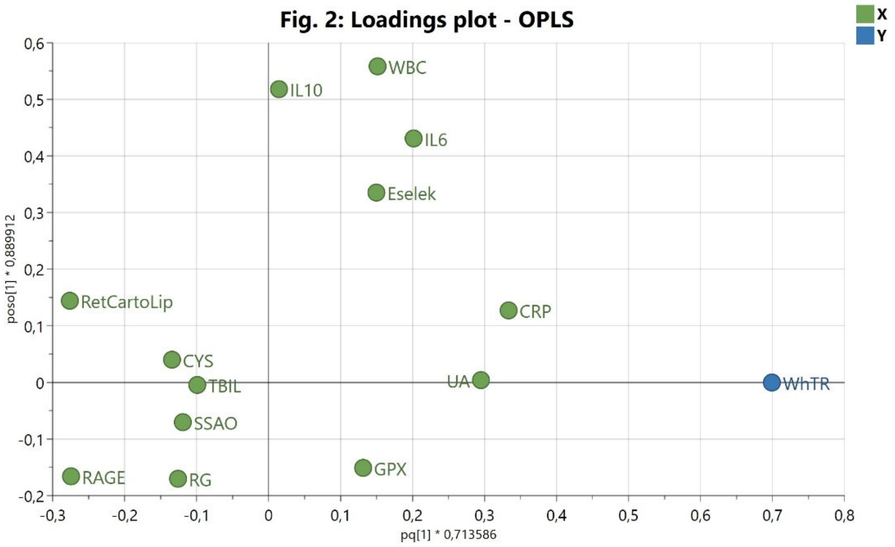

Loading scatter plot displays the relationship between independent variables (green circles) and a dependent variable (blue circle) in the whole cohort. X: independent variables (green); y: dependent variable – WhTR, waist/height ratio, (blue); RetCartoLip, retinol + carotenoids (=ß-carotene + xanthophyll + lycopene)/(total cholesterol + triacylglycerols); SSAO, semicarbazide-sensitive aminoxidase; RAGE, soluble receptor for advanced glycation end products; RG, reduced glutathione (GSH); CYS, cysteine; GPX, glutathione peroxidase; Eselek, soluble E-selectin; TBIL, total bilirubin; IL10, interleukine-10; IL6, interleukine-6; UA, uric acid; WBC, white blood cell counts; CRP, C-reactive protein

Supplementary Figure S3. Loading scatter plot – multivariate regression of independent variables - markers of oxidative status and inflammation on fasting plasma glucose using the orthogonal projection to latent structures model

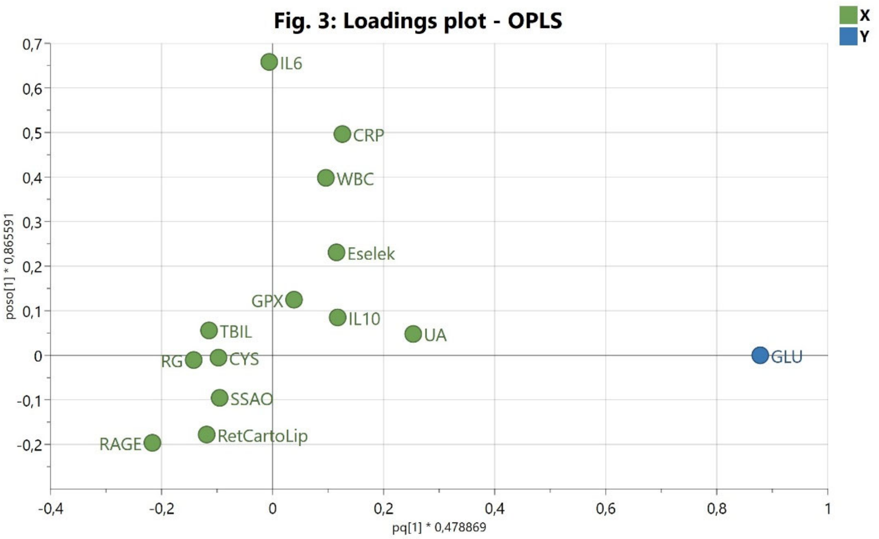

Loading scatter plot displays the relationship between independent variables (green circles) and a dependent variable (blue circle) in the whole cohort. X: independent variables (green); y: dependent variable – fasting plasma glucose, GLU, (blue); RetCartoLip, retinol + carotenoids (=ß-carotene + xanthophyll + lycopene)/(total cholesterol + triacylglycerols); SSAO, semicarbazide-sensitive aminoxidase; RAGE, soluble receptor for advanced glycation end products; RG, reduced glutathione (GSH); CYS, cysteine; GPX, glutathione peroxidase; Eselek, soluble E-selectin; TBIL, total bilirubin; IL10, interleukine-10; IL6, interleukine-6; UA, uric acid; WBC, white blood cell counts; CRP, C-reactive protein

Supplementary Figure S4. Loading scatter plot – multivariate regression of independent variables - markers of oxidative status and inflammation on triacylglycerols using the orthogonal projection to latent structures model

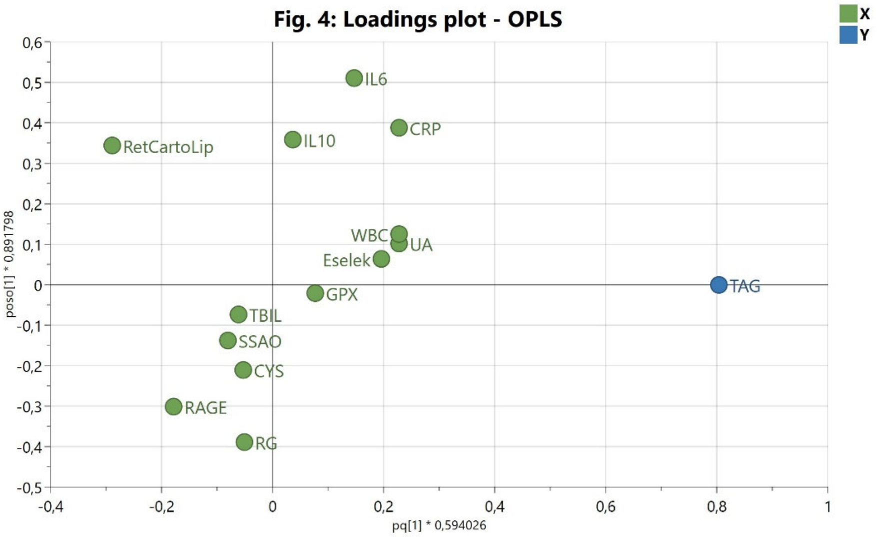

Loading scatter plot displays the relationship between independent variables (green circles) and a dependent variable (blue circle) in the whole cohort. X: independent variables (green); y: dependent variable – TAG, triacylglycerols, (blue); RetCartoLip, retinol + carotenoids (=ß-carotene + xanthophyll + lycopene)/(total cholesterol + triacylglycerols);

SSAO, semicarbazide-sensitive aminoxidase; RAGE, soluble receptor for advanced glycation end products; RG, reduced glutathione (GSH); CYS, cysteine; GPX, glutathione peroxidase; Eselek, soluble E-selectin; TBIL, total bilirubin; IL10, interleukine-10; IL6, interleukine-6; UA, uric acid; WBC, white blood cell counts; CRP, C-reactive protein

Supplementary Figure S5. Loading scatter plot – multivariate regression of independent variables - markers of oxidative status and inflammation on high-density lipoprotein cholesterol using the orthogonal projection to latent structures model

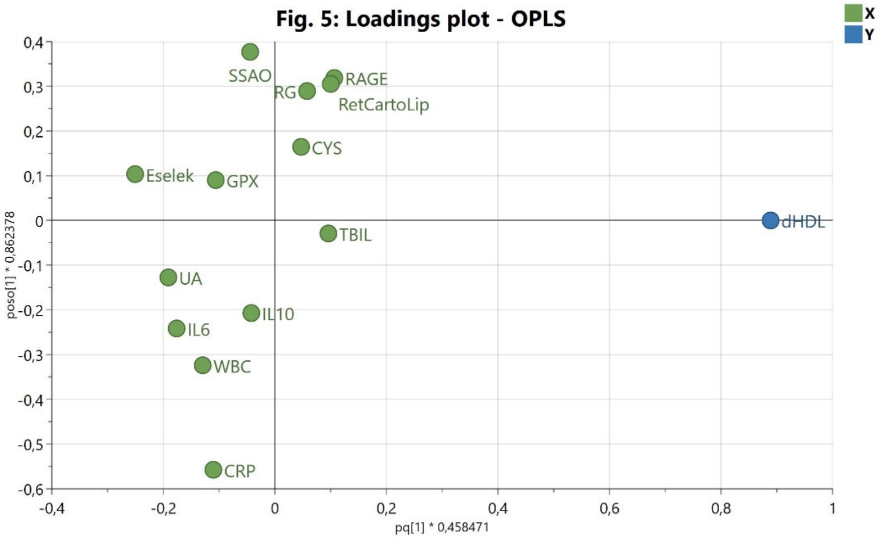

Loading scatter plot displays the relationship between independent variables (green circles) and a dependent variable (blue circle) in the whole cohort. X: independent variables (green); y: dependent variable – dHDL, (direct) high-density lipoprotein cholesterol (blue); RetCartoLip, retinol + carotenoids (=ß-carotene + xanthophyll + lycopene)/(total cholesterol + triacylglycerols); SSAO, semicarbazide-sensitive aminoxidase; RAGE, soluble receptor for advanced glycation end products; RG, reduced glutathione (GSH); CYS, cysteine; GPX, glutathione peroxidase; Eselek, soluble E-selectin; TBIL, total bilirubin; IL10, interleukine-10; IL6, interleukine-6; UA, uric acid; WBC, white blood cell counts; CRP, C-reactive protein

Supplementary Figure S6. Loading scatter plot – multivariate regression of independent variables - markers of oxidative status and inflammation on systolic blood pressure using the orthogonal projection to latent structures model

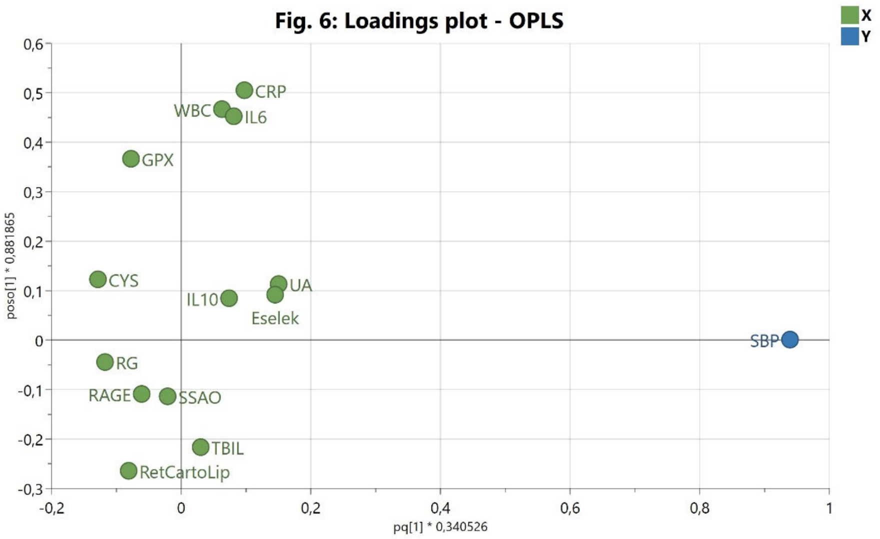

Loading scatter plot displays the relationship between independent variables (green circles) and a dependent variable (blue circle) in the whole cohort. X: independent variables (green); y: dependent variable – systolic blood pressure, SBP, (blue); careoidstoLip, carotenoids (=ß-carotene + xanthophyll + lycopene)/(total cholesterol + triacylglycerols); SSAO, semicarbazide-sensitive aminoxidase; RAGE, soluble receptor for advanced glycation end products; RG, reduced glutathione (GSH); CYS, cysteine; GPX, glutathione peroxidase; Eselek, soluble E-selectin; TBIL, total bilirubin; IL10, interleukine-10; IL6, interleukine-6; UA, uric acid; WBC, white blood cell counts; CRP, C-reactive protein.
